# Recent Advances in Ovarian Cancer: Therapeutic Strategies, Potential Biomarkers, and Technological Improvements

**DOI:** 10.3390/cells11040650

**Published:** 2022-02-13

**Authors:** Salima Akter, Md. Ataur Rahman, Mohammad Nazmul Hasan, Hajara Akhter, Priya Noor, Rokibul Islam, Yoonhwa Shin, MD. Hasanur Rahman, Md. Shamim Gazi, Md Nazmul Huda, Nguyen Minh Nam, Jinwook Chung, Sunhee Han, Bonglee Kim, Insug Kang, Joohun Ha, Wonchae Choe, Tae Gyu Choi, Sung Soo Kim

**Affiliations:** 1 Department of Biochemistry and Molecular Biology, School of Medicine, Kyung Hee University, Seoul 02447, Korea; salima_2015@buhs.ac.bd (S.A.); jac03032@khu.ac.kr (Y.S.); sunheehan@khu.ac.kr (S.H.); iskang@khu.ac.kr (I.K.); hajh@khu.ac.kr (J.H.); wchoe@khu.ac.kr (W.C.); 2 Biomedical Science Institute, Kyung Hee University, Seoul 02447, Korea; 3 Department of Medical Biotechnology, Bangladesh University of Health Sciences, Dhaka 1216, Bangladesh; priyabge1509@gmail.com; 4 Department of Pathology, College of Korean Medicine, Kyung Hee University, Seoul 02447, Korea; rahman23@khu.ac.kr (M.A.R.); bongleekim@khu.ac.kr (B.K.); 5 Korean Medicine-Based Drug Repositioning Cancer Research Center, College of Korean Medicine, Kyung Hee University, Seoul 02447, Korea; 6 Global Biotechnology & Biomedical Research Network (GBBRN), Department of Biotechnology and Genetic Engineering, Faculty of Biological Sciences, Islamic University, Kushtia 7003, Bangladesh; 7 Pristine Pharmaceuticals, Patuakhali 8600, Bangladesh; nobinbge@gmail.com; 8 Biomedical and Toxicological Research Institute, Bangladesh Council of Scientific and Industrial Research (BCSIR), Dhaka 1205, Bangladesh; hajara@bcsir.gov.bd; 9 Department of Biotechnology and Genetic Engineering, Faculty of Biological Sciences, Islamic University, Kushtia 7003, Bangladesh; mrislam@btge.iu.ac.bd; 10 Department of Biochemistry, College of Medicine, Hallym University, Chuncheon 24252, Korea; 11 Department of Biomedical Science, Graduate School, Kyung Hee University, Seoul 02447, Korea; cck608@khu.ac.kr; 12 Department of Biotechnology and Genetic Engineering, Bangabandhu Sheikh Mujibur Rahman Science and Technology University, Gopalganj 8100, Bangladesh; hasanurrahman.bge@gmail.com; 13 Biotechnology and Genetic Engineering Discipline, Khulna University, Khulna 9208, Bangladesh; shamimgazibge@ku.ac.bd; 14 Department of Biochemistry and Molecular Biology, UAMS Winthrop P. Rockefeller Cancer Institute, University of Arkansas for Medical Sciences UAMS, Little Rock, AR 72205, USA; mnhuda@uams.edu; 15 Research Center for Genetics and Reproductive Health, School of Medicine, Vietnam National University Ho Chi Minh City, Linh Trung Ward, Thu Duc District, Ho Chi Minh City 71308, Vietnam; nmnam@medvnu.edu.vn

**Keywords:** ovarian cancer, angiogenesis, technology advances, molecular insight, therapeutic strategies and targets

## Abstract

Aggressive and recurrent gynecological cancers are associated with worse prognosis and a lack of effective therapeutic response. Ovarian cancer (OC) patients are often diagnosed in advanced stages, when drug resistance, angiogenesis, relapse, and metastasis impact survival outcomes. Currently, surgical debulking, radiotherapy, and/or chemotherapy remain the mainstream treatment modalities; however, patients suffer unwanted side effects and drug resistance in the absence of targeted therapies. Hence, it is urgent to decipher the complex disease biology and identify potential biomarkers, which could greatly contribute to making an early diagnosis or predicting the response to specific therapies. This review aims to critically discuss the current therapeutic strategies for OC, novel drug-delivery systems, and potential biomarkers in the context of genetics and molecular research. It emphasizes how the understanding of disease biology is related to the advancement of technology, enabling the exploration of novel biomarkers that may be able to provide more accurate diagnosis and prognosis, which would effectively translate into targeted therapies, ultimately improving patients’ overall survival and quality of life.

## 1. Introduction

Ovarian cancer (OC) is the presence of abnormal cells that initially grow in the ovary and then reproduce out of control, which can form a tumor malignancy when they spread into the surrounding tissues [1,2]. Ovaries are made up of three types of cells, and each cell can develop into diverse types of tumors. Approximately 90% of ovarian cancers have been found to be of epithelial origin [3], including high-grade and low-grade serous carcinoma and clear cell, endometrioid, and mucinous carcinoma, while 7% of OCs have been shown to be stromal types, and OCs from germ cell tumors are found only rarely [1]. It has been found that there are frequently warning symptoms and signs for OC; however, the earliest symptoms are unclear and hard to detect due to shared gastrointestinal, genitourinary, and gynecological conditions [4]. A number of barriers to the treatment of the disease exist [5,6]. Despite the early high rates of response to initial chemotherapy and radical surgery for about 70% of patients with relapses and intermediate progression-free 12- to 18-month survival, long-term survival remains poorly understood, with a high risk of reappearance [7]. Additionally, chemotherapeutic treatments for OC have an undesirable impact on quality of life because of their severe side effects, including fatigue, arthralgia, and neurotoxicity [8,9]. Therefore, understanding the biology of heterogeneous OCs is vital for exploring the disease’s mechanisms more accurately [4]. Potential therapeutic targets for the management of OC are being explored, such as intrinsic signaling pathways, angiogenesis, hormone receptors, and immunologic factors. 

Bevacizumab, the most-studied anti-VEGF-targeted therapy inhibiting angiogenesis in the tumor microenvironment, holds great promise for OC treatment, but redundant angiogenic pathways make the drug show only modest efficacy [5,6,10,11]. Meanwhile, there has been a surge in clinical trials with several drug candidates that precisely target signal enzymes, which may induce apoptosis and autophagy, targeting the inhibition of angiogenesis in site-specific OC cells [12,13,14,15]. However, to understand the disease’s pathophysiology, it is essential to thoroughly investigate the regulatory mechanisms in terms of the different molecular layers and time intervals, which may clearly demonstrate the disease dynamics [16,17]. Indeed, the use of molecular profiling for patients with OC may provide effective strategies for treating the disease. Using multi-omics data, it may be possible to gain a comprehensive understanding of the tumor’s biology, which could make it feasible to discover prognostic biomarkers or predictors to facilitate the early diagnosis and prognostic prediction of aggressive and advanced OC, which could ultimately help in treatment decisions [4,18,19].

Drug delivery or co-delivery systems represent another crucial approach for OC treatment. Single targeted drugs or multiple targeted agents have been engineered for drug-delivery systems that realize drug release more effectively and reduce toxicity. The present study attempted to evaluate the recent understanding of ovarian cancer associated with signaling mechanisms, targeted therapeutic strategies, and potential drug delivery systems. In particular, the interplay between technological advancement and the management of this heterogeneous disease from diverse perspectives is highlighted.

## 2. Targeting Numerous Signaling Pathways of Ovarian Cancer

Surgery and chemoradiotherapy are the most frequently used treatment options for ovarian cancer (OC) [20]. However, severe side effects have been associated with chemo- and radiotherapy (RT), while the only minor therapeutic benefit from RT eventually leads to succumbing to the disease and poor survival outcomes [21]. Hence, targeting specific signaling pathways would be a promising molecular approach to ovarian cancer therapy in terms of inhibiting tumor growth, cell invasion or migration, and metastasis. It was found that seven major signaling pathways are commonly upregulated in ovarian cancers (> 50%): the PI3K/AKT/mTOR, Jak/STAT, Src, lysophosphatidic acid (LPA), NF-κB, PKCι, and Mullerian inhibitory substance receptor signaling pathways have shown high levels of mutation and/or hyperactivation strongly associated with aggressive phenotypes and advanced disease stages, leading to poor prognosis for the disease [4,22]. In this section, we briefly describe some signaling pathways related to tumorigenesis and metastasis that may be potentially targetable and provide information regarding novel inhibitors currently in clinical trials.

### 2.1. PI3K/AKT/mTOR Pathway

**Figure 1:**
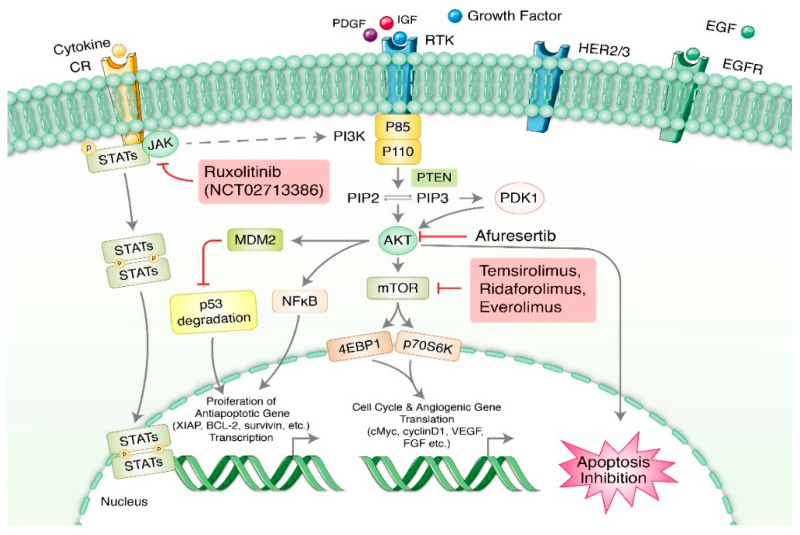
PI3K/Akt/mTOR signaling pathway. This pathway is upregulated in ovarian cancer by either (i) receptors of upstream growth factors and ligand stimulation, (ii) indirect activation via cross-talk with JAK/STAT signaling, or (iii) intrinsically via activation of amplified/mutated PI3K or amplification of Akt isoform, or deletion/inactivation in tumor-suppressor protein PTEN. Afuresertib, an Akt inhibitor, is safely used in platinum-resistant ovarian cancer. Most frequently studied mTOR inhibitors in completed OC phase II clinical trials are temsirolimus, ridaforolimus, and everolimus. Ruxolitinib, a JAK inhibitor, is already FDA approved for treatment of polycythemia vera.

Phosphoinositide 3-kinase (PI3K)/AKT/mammalian target of rapamycin (mTOR) signaling is one of the most important pathways controlling cell growth, proliferation, differentiation, and survival [23]. The pathway is regulated by multiple ligands, such as growth factors (IGF, EGF, TGF, and others), receptor tyrosine kinases (IGF-1R, FGFR, HER2, EGFR, and PDGFR), and various membrane receptors [24,25,26]. Indeed, mutations in several components of the pathway are very common in most human cancers, including subtypes of OC [27]. It has been shown that the aberrant expression and activation of AKT (pAKT) is strongly correlated with poor progression-free and overall survival in epithelial OC [28]. Whole-genome sequencing analysis revealed that gene breakage frequently inactivates the tumor-suppressive ability of RB1, PTEN, NF1, and RAD51B in high-grade serous ovarian cancer, resulting in acquired chemoresistance [29]. In particular, OC stemness (CSC), the key regulatory factor of aggressive cancer, is directly modulated by PI3K/PTEN/AKT signaling, causing CSC enrichment, CSC phenotyping maintenance, and multidrug resistance (MDR) [30,31], which leads to abnormal cell proliferation and cancer metastasis through epithelial–mesenchymal transition [32]. The well-studied mTOR inhibitors for OC include temsirolimus, ridaforolimus, and everolimus, for which phase II clinical trials have been completed [13]. In recent in vitro and in vivo studies, SPR965, a dual inhibitor of PI3K and mTORC1/2, has been proven to have antitumorigenic activity in diverse solid tumors, including serous ovarian cancer. However, further clinical trials are needed before it can be recommended as a novel targeted therapeutic agent [33]. Afuresertib, an Akt inhibitor, showed a satisfactory safety profile in platinum-resistant OC in a phase I study, and the drug NCT04374630 is under investigation for use in combined therapy with paclitaxel in platinum-resistant OC in a phase II trial (Figure 1).

### 2.2. JAK/STAT Signaling Pathway

The JAK/STAT pathway is a crucial signaling pathway that is abnormally activated in OC, and its constitutive activation is strongly related to tumor progression and poor prognosis for the disease [34]. Hyperstimulation of this pathway has also been found in other cancers, including breast, gastric, lung, prostate, and hematopoietic malignancies [35,36,37]. JAK/STAT pathway-mediated tumor progression is mainly due to the expression of a variety of proteins and cytokines involved in cellular proliferation, stemness and self-renewal, survival, and evasion of antitumor immunity [37,38]. Studies have found that more than 50 cytokines and growth factors are responsible for this pathway initiating hematopoiesis, inflammation, and immune suppression in the tumor microenvironment [38]. STAT is a key driver of immunosuppression through triggering the production of immune checkpoint genes (e.g., PD1, PD-L1, PD-L2, and CTLA-4) [39], promoting radio- and/or chemoresistance and the failure of targeted immunotherapies [22]. 

JAK inhibitors have been found to be essential in the treatment of cancer in recent years. Many JAK inhibitors have demonstrated efficacy in clinical settings, and a number of inhibitors/analogs are currently being studied. Ruxolitinib is a JAK inhibitor already approved by the FDA for the treatment of polycythemia vera; in preclinical studies, it was found that the drug reduced cell viability in OC [40]. NCT02713386 is being investigated for use in combination therapy with paclitaxel and carboplatin in stage III–IV OC in a completed phase I/II trial [13]. AZD1480, a small-molecule JAK inhibitor, was demonstrated to suppress OC growth in a mouse model via cascade inhibitory effects on STAT3 phosphorylation, DNA binding, migration, and the adhesion of cultured ovarian cancer cells [41]. AH057 may effectively block the pathway by inhibiting the function of JAK1/2 kinase, resulting in increased cell cycle arrest and apoptosis, and impaired tumor progression and invasion, as shown in vitro and in vivo [42]. In CSC, CYT387 administration with paclitaxel was shown to suppress JAK2/STAT3 activity and paclitaxel-induced Oct4 and CD117 (CSC-like marker) expression in a mouse xenograft model, suggesting the development of CSC-targeted therapy [43]. Higher levels of aldehyde dehydrogenase (ALDH), a characteristic feature of endometrial cancer progenitor and stem cells, upregulates IL-6 and signal transducer CD126 and GP130 expression, while the blockade of the IL6 receptor dramatically suppresses downstream effector IL6/JAK1/STAT3 signaling, eventually reducing tumor cell growth [44]. Taking all the data together, the continuous activation of the JAK/STAT pathway is certainly implicated in many types of human malignancies, while the potential effect of JAK inhibitors on cancer development remains a source of concern [45]. Therefore, the safety and benefits of JAK inhibitors still need to be determined.

### 2.3. Wnt/β-Catenin Pathway

The interest in Wnt signaling began in 1982 and has steadily increased due to the extreme renewal, proliferation, and differentiation properties of CSCs, thus showing an important role for them in tumorigenesis and therapy resistance in many malignancies [46]. Wnt signaling exemplifies several pathways, such as Notch–Delta, Sonic–Hedgehog, Hippo, and transforming growth factor β (TGF-β)/bone morphogenetic protein (BMP), which are directly implicated in developmental and evolutionary processes [47], thereby facilitating its widespread activity. Wnt signaling seems to regulate tumorigenesis in ways that are both β-catenin-dependent (canonical, primarily for cell proliferation) and β-catenin-independent (noncanonical, controlling cell polarity and movement) [48]. Although Wnt signaling has been linked to the incidence and progression of ovarian cancer [49], its possible consequences in ovarian cancer are still being investigated.

Mutations in the components of the Wnt pathway are causal factors for multiple growth-associated pathologies in cancer [47]. A number of possible mechanisms are involved in Wnt pathway hyperactivation, including the upregulation of ligands and receptors, the downregulation of the Wnt/beta-catenin pathway inhibitors, and altered protein function, which in turn control the interaction between beta-catenin and E-cadherin or beta-catenin and TCF. These abnormalities have been noted in EOC, especially in the high-grade serous subtype [50]. Furthermore, the involvement of several noncoding RNAs (IncRNAs, miRNAs, and circRNAs) in regulating beta-catenin signaling in EOC has recently been demonstrated [51]. Wnt signals regulate the cell cycle at several points. In endometrial and mucinous subtypes of EOC, mutations have been observed in, for example, the CTNNB1, AXIN, and APC genes [50]. The crucial role of the Wnt pathway in OC development, progression, angiogenesis, metastasis, and chemoresistance is supported by its strong CSC (cancer stem cell) self-renewal, EMT (epithelial–mesenchymal transition), and invasion capabilities and tumor immunity suppression [52]. Apart from tumorigenesis, there is a direct impact of the Wnt signaling pathway on immune responses. Recently, several cancer-specific inhibitors of this signaling pathway have been identified, including WNT974, which increases antitumor immunity in ovarian cancer [53]. Thus, β-catenin may be a promising therapeutic target in chemoresistance subtypes of EOC with CSCs.

### 2.4. Apoptotic Signaling Pathway’

Apoptosis is a characteristic and orderly energy-mediated biochemical cellular suicide process that maintains homeostatic equilibrium between the proportion of cell death and cell formation in multicellular creatures [54,55]. It is well evidenced that apoptosis induction acts as a hallmark barricade to cancer development [56,57,58]. The B-cell lymphoma-2 (BCL-2) family and inhibitors of apoptotic proteins (IAPs) are the predominant components of intrinsic apoptotic pathway induction through caspase activation, which regulates mitochondrial membrane permeabilization through apoptotic switching [59]. Alternatively, the extrinsic apoptotic pathway triggers tumor necrosis factor (TNF)-related apoptosis-inducing ligand (TRAIL) to the cell surface receptor signaling cascade [60]. Several studies imply that both signaling cascades may be activated simultaneously to induce apoptosis in human ovarian cancer [61,62]. In particular, it has been proposed that apoptosis induction is broadly mediated by caspase-3 pathway activation, which has been established by increased sensitivity to paclitaxel using adenoviral type 5 E1A in human HER-2/neu-overexpressing ovarian cancer SKOV3.ip1 cells. In this pathway, caspase-3 executes the proteolytic cleavage of cellular proteins to progress apoptosis [63]. 

Enzastaurin (LY317615.HCl), a radiosensitizing, ATP-competitive, discriminating protein kinase C beta (PKC-beta) inhibitor, is an alternative drug that inhibits tumor cell growth through the upregulation of caspase-3 and caspase-9′s proapoptotic activity [62]. Among different analyses, a combination treatment with enzastaurin and pemetrexed was shown to cause apoptosis induction in chemotherapy-resistant ovarian cancer HEY cells, controlling phosphorylated GSK3β and inhibiting mitogen-activated protein kinase ERK-1/2 (extracellular signal-regulated kinase)-mediated cell growth [64]. In addition, a current study reveals that metformin induces an apoptotic pathway in OVCAR-3 and OVCAR-4 cell lines in an AMP-activated protein kinase (AMPK)-independent manner, resulting in S- and G2/M-phase cell cycle arrest. Metformin may also induce apoptosis by downregulating Bcl-2 and Bcl-xL protein expression and caspase 3/7 activation, and augmenting Bax and Bad expression in human OVCAR-3 and OVCAR-4 cell lines. Furthermore, metformin-induced apoptosis is augmented by the addition of cisplatin without modulating the appearance of Bcl-2 proteins in the OVCAR-3 cell line, although BcL-2 was expressed in the OVCAR-4 cell line [65].

**Figure 2:**
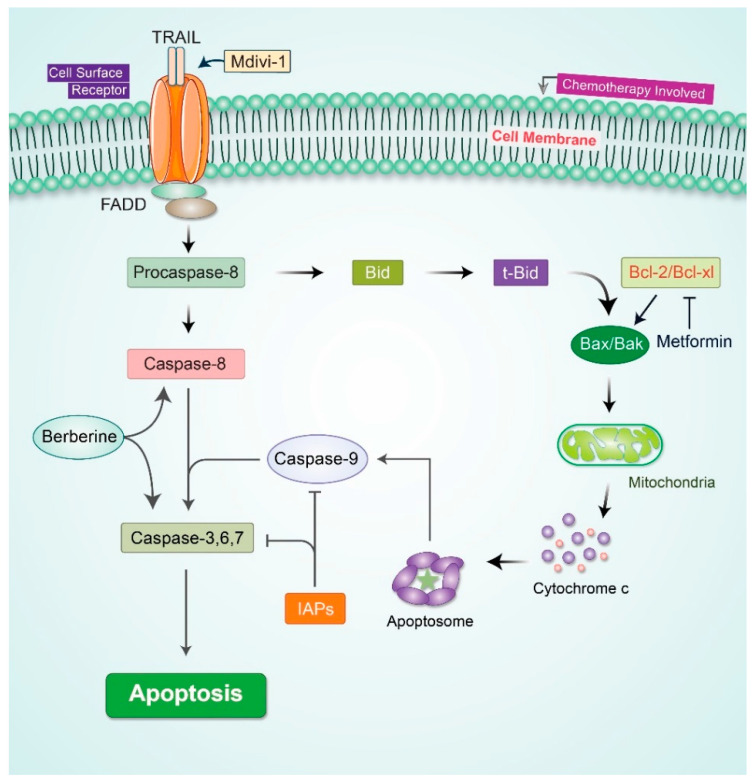
Apoptosis signaling in cancer cells. Metformin-induced apoptotic pathway in ovarian cancer cell lines stimulates AMP-activated protein kinase (AMPK)-independent apoptotic pathway. Berberine activates caspase-8 and caspase-3-mediated apoptotic pathway. Mdivi-1 stimulates TRAIL-induced extrinsic pathway.

Resveratrol, a small polyphenol compound, increases apoptosis induction by activating it in an AMPK-dependent manner, and activates caspase 3, which leads to the inhibited expression and activation of mTOR, a downstream signaling target of AMPK, in ovarian cancer cells [66]. Moreover, TRAIL has been reported as an alternative therapeutic target for ovarian cancer management, although the targeted restriction of tyrosine kinase family proteins (PYK2 and FAK) and BCL-XL works synergistically and increases apoptosis in human ovarian carcinoma cell lines. The study revealed that the mitochondrial division inhibitor-1 (mdivi-1) increases the sensitivity of ovarian cancer cells to cell surface ligands such as FAS, TRAIL, and TNF-alpha [67]. A recent study demonstrated that berberine (BBR), a potent anticancer drug, combined with cisplatin (DDP) enhanced apoptosis by inhibiting PCNA and Ki67 expression and upregulating caspase-3, caspase-8, RIPK3, and MLKL expression and activation in the OVCAR-3 and POCCL ovarian cancer cell lines [68] (Figure 2).

## 3. Autophagy Modulation in Ovarian Cancer Management

Autophagy is a self-digestion process that assists in maintaining cellular homeostasis by recycling unwanted or damaged toxic cellular organelles in cells [69,70]. The modulation of autophagy has been implicated in regulating several cancers [71,72]. It has been suggested that autophagy is an important function in ovarian cancer via the expression of autophagy-related proteins, which comprise the microtubule-associated proteins light chain (LC-3), beclin-1, and p53 [12]. Beclin-1 is a tumor-suppressor protein that has an essential checkpoint role in apoptosis and autophagy in tumor cells [73]. Beclin-1 expression has been found to be downregulated in ovarian cancers compared to benign lesions [74], suggesting the predictive potential of the beclin-1 protein in OC. Furthermore, the cytoplasmic localization of p53 mutants has been shown to prevent autophagy [75]. Additionally, Bcl-2 expression was found to prevent autophagy by interacting with beclin-1, and the overexpression of mutant p53 protein may impact autophagy in ovarian cancer cells [76]. Later, the p53-mediated regulation of autophagy was validated in a clinical study [77].

**Figure 3:**
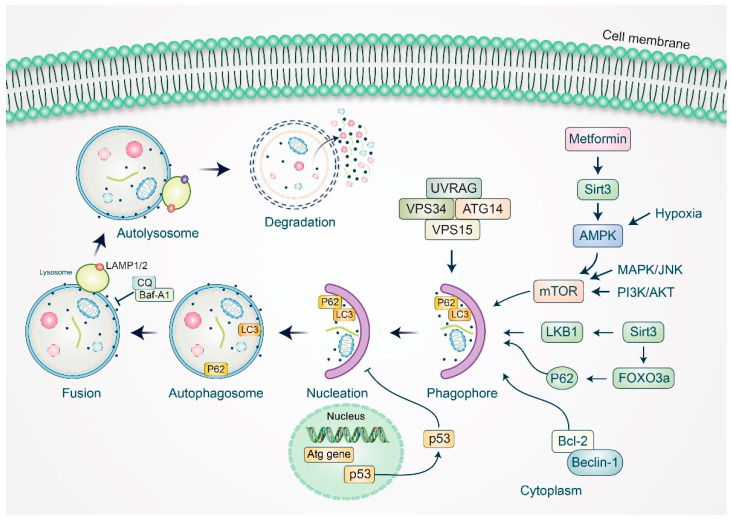
Modulation of autophagy signaling in relation to Sirt3 and autophagy in ovarian cancer. Metformin-mediated Sirt3 overexpression triggers AMPK, which increases activation of LC3. Sirt3 is also involved in autophagy regulation through MAPK/JNK/mTOR autophagy pathway. Several autophagy-related genes, such as beclin-1, p62, LKB1, and VPS34 complex, stimulate autophagy initiation. Sirt3 also activates FOXO3a, which subsequently activates p62 and autophagy. Transcription factor p53 activates and promotes synthesis of autophagy proteins, and high cytoplasmic levels of p53 may result in inhibition of autophagosome formation.

Aplasia Ras homolog member I (ARH1), another protein, has been found to be upregulated in autophagy via the mTOR-dependent pathway, which activates autophagy-mediated dormancy [78]. In approximately 70% of cases of ovarian cancer, PI3K/AKT/mTOR pathways have been shown to be constitutively triggered by autophagy, which has been considered to be a therapeutic target of ovarian cancer [79]. It was reported that a specific PI3K inhibitor, LY294002, given as treatment for ovarian cancer in an established mouse model, prevented ovarian cancer cell proliferation [80]. Additionally, the cellular cytotoxic effects of novel chemotherapeutic agents were shown to be efficiently improved through cotreatment with a noncompetitive AKT inhibitor, TAS-117, in in vivo models of ovarian cancer [81]. Sirtuin 3 (Sirt3), a member of the sirtuin protein family, performs an essential function in maintaining ovarian cancer intracellular homeostasis in a close mutual monitoring relationship, as well as autophagy. Studies showed that metformin-mediated Sirt3 overexpression encouraged mitochondrial dysfunction and apoptosis via the activation of AMPK in ovarian cancer cells [82]. In addition, Sirt3 may similarly control autophagy through glutathione S-transferase and JNK-mediated autophagy pathways, and Sirt3 knockdown was shown to relieve S1-induced apoptosis in ovarian cancer cells [83] (Figure 3).

## 4. Novel Treatment Strategies for Epithelial Ovarian Cancer (EOC)

The identification of novel therapeutic targets has been linked to better prognosis in ovarian cancer (OC). Advancements in the understanding of ovarian cancer biology have resulted in the development of numerous molecular targets, including growth factor receptors, cell cycle regulators, signal transduction pathways, and angiogenic mechanisms. The molecularly targeted agents possess higher selectivity and lower toxicity than conventional chemotherapy [84]. Major therapeutic targets used alone or in combination with cytotoxic drugs for OC treatment and new drugs in clinical trials are reviewed in this section.

### 4.1. Therapeutic Approaches and Targets in Ovarian Cancer

Given the enormous number of potential epithelial ovarian cancer treatments, it is useful to review the pathobiology of the disease to find relevant targets. The drug targets telomerase, HER2, AKT EGF-R, VEGF-R, and p53 are currently being studied in clinical trials [13,85]. Some specific targets are found only in OC, while some found in a wide variety of cancers [85,86] are briefly discussed. 

### 4.2. Angiogenesis and VEGF Signaling Pathway

Angiogenesis is the process of forming new blood vessels, which enables nutrients and oxygen to enter the surrounding tissues, thus promoting tumor cell proliferation, invasion, and metastasis [87,88]. The growth of blood vessels or new capillaries starts with vasodilation, increased vascular permeability, stromal disintegration, and endothelial cell proliferation and migration [89,90]. Researchers have discovered that receptor tyrosine kinases (RTKs), VEGF and its receptor (VEGFR), and Flk-1/KDR RTK play key roles in pathological angiogenesis, particularly tumor neovascularization [91]. An immediate impact on tumor growth is observed (slowdown or stoppage) when the VEGF signaling pathway is blocked or inhibited [92]. This insight into the mechanism of angiogenesis led to the establishment of several treatment methods targeting the VEGF pathway. 

Bevacizumab is an anti-VEGF antibody and the most studied VEGF-targeting therapy for ovarian cancer [93,94]. The best response of the drug has been found in recurrent ovarian cancer, and it can be administered alone or with chemotherapy [95,96,97]. Current ovarian cancer clinical trials with bevacizumab show promising results (PFS) in two major first-line studies, ICON7 [98] and GOG 218 [99]. Along with carboplatin/paclitaxel, the GOG study uses bevacizumab as part of a triplet to treat patients with minimal cytoreduction of ovarian cancer [100]. Other potential VEGF-targeting medicines, including soluble decoy VEGF receptors such as aflibercept (VEGF TRAP) [101] and VEGF kinase inhibitors such sunitinib (SU11248, Sutent, Pfizer), have shown significant treatment benefit in EOC patients [14].

### 4.3. ErbB Family Kinases

The EGF family of RTKs, also known as ErbB or HER receptors, has been widely investigated in pharmacological research targeting human cancer. Numerous hypotheses have been suggested for HER2-mediated cell transformation through multiple mechanisms, such as EGFR and ErbB-3 interaction, which exhibit tyrosine phosphorylation and the activation of a cytoplasmic signaling pathway, while ErbB1 and ErbB2 homodimers transform fibroblasts using differential signaling [102,103]. Trastuzumab (Herceptin), a targeted monoclonal antibody for ErbB2, is approved for treating ErbB2 1 breast cancer. According to the GOC study, trastuzumab had limited action in ovarian cancer [104]. A partial but long-lasting response was observed when combination therapy with trastuzumab–pertuzumab was used in a young woman with high-grade serous ovarian cancer (FIGO stage IV) [15]. Furthermore, a number of EGF-R targeting agents are currently in clinical trials [105,106,107], while some agents have shown exciting antitumor performance in CRC-based xenograft models and cell lines, such as cabozantinib, and are awaiting clinical trials [10]. Other receptor-binding inhibitors, such as cetuximab, work differently from the TKIs gefitinib and erlotinib. However, erlotinib and gefitinib alone show poor response rates (5–10%) in EOC [11,108] owing to PI3K-pathway-mediated tumor resistance through p38 MAPK activation and the following DNA repair [109]. Thus, targeting of EGFR, along with inhibition of p38 MAPK or DNA repair, may improve the efficacy of EGFR mediated treatment in ovarian cancer.

### 4.4. Ansamycins and HSP90 Degradation 

Benzoquinone ansamycin 17-allylamino-17-demethoxygeldanamycin (17-AAG) is an early described tyrosine kinase inhibitor that interacts with HSP90, leading to the proteasomal degradation of Hsp90-targeted proteins [110,111]. Many biological functions of 17-AAG are common with those of its parent compound geldanamycin (GA), including the ability to inhibit the growth of tumor cells [112]. It has been shown that Hsp90 originating from tumor cells has a 100-fold higher affinity to bind with 17-AAG than Hsp90 from normal cells, and also a strong affinity for oncogenic signaling proteins such as HER-2/ErbB2, Akt, Raf-1, mutated p53, and Bcr-Abl, emphasizing it as an attractive candidate for new treatment options in OC. For example, ErbB2 appears to be a potential target, because high ErbB2-expressing cells are more susceptible to ansamycin-induced growth inhibition at minimal doses. Surprisingly, this effect seems to be linked to ErbB3- and PI3K/AKT-dependent pathways [113]. Ansamycins are known to have a strong affinity for the AKT protein. For AKT to remain stable, HSP90 needs to be linked to it, and the addition of HSP90 inhibitors results in a gradual decrease in AKT function [114,115]. Thus, the PI3K–AKT signaling pathway is highly active in the progression of OC, and combination therapy of 17-AAG with cisplatin or taxol may enhance cell apoptosis via the inhibition of PI3K/Akt signaling. In addition, the combination of olaparib and 17-AAG may increase drug sensitivity in HR-proficient EOC and reverse multidrug resistance [116], suggesting the rational use of 17-AAG in ovarian cancer.

### 4.5. 26S Proteosome Inhibition with PS341 (Bortezomib)

The activity of the proteasome directly represents a promising therapeutic strategy for cancer. PS341 (bortezomib), a dipeptide boronic acid derivative, prevents protein degradation by the reversible inhibition of the 20S proteasome. Cyclins (CDKs) and IkB proteins, which are corepressors of nuclear factor-kappa B (NF-κB) activation, seem to be the prospective targets. The inhibition of IkB degradation reduces NF-κB transcription factor activity [117]. Although NF-kB appears to have a strong antiapoptotic function, the use of PS-341 and NF-κB blockers tends to increase chemotherapy-induced apoptosis.

### 4.6. Tubulin-Targeting Molecules

Anticancer drugs, including taxanes and vinca alkaloids, which are directed against microtubules, have long been used as first-line drugs for breast cancer and a wide range of other cancers, including ovarian, prostate, head and neck, and lung cancers [86]. Polyglutamated paclitaxel (CT2103), a cytotoxic agent, was found to have fewer side effects and better treatment responses than paclitaxel in phase III clinical trials [118]. Compared to the original paclitaxel, this new formulation has a decreased risk of hypersensitive side effects and can be administered more quickly. Indeed, it shows taxane-like efficacy in recurrent OC, with a response rate of 23% in individuals who have received limited prior therapy; however, oral treatment results in low bioavailability [119].

### 4.7. Ovarian Cancer-Specific Targets: MUC16/CA125

For more than two decades since its discovery, CA125 antigen has been permitted for clinical use for the OC screening of high-risk women in the US. Later, it was suggested as a predictive marker in preinvasive OC [120]. Although it has limited sensitivity and specificity, the CA125 antigen is strongly related to epithelial OC. The diagnostic performance of this biomarker has been useful in primary care, especially in women ≥ 50 years old [121]. Lloyd and colleagues identified the gene that encodes the CA125 antigen, which was subsequently called MUC16 [122]. The affinity for the binding of CA125 antigen with the murine monoclonal antibody Mab-B3.13 (also known as OvaRex) is strong. Thus, CA125-targeted murine antibodies have been employed as potential therapeutic agents. In a phase I/II clinical trial, patients with recurrent OC developed immunity, such as antibodies and T cells, to oregovomab and CA125 given as third-line therapy, and anti-idiotype antibodies were found in 66% of patients [123,124]. This suggests that vaccination using specific anti-idiotypic antibodies could ameliorate the survival benefit for patients with few side effects in recurrent OC. Therefore, the application of the noninvasive immunotherapy in combination chemotherapy may be a potential therapeutic strategy for improved survival in OC [125].

## 5. Drug-Delivery System for Ovarian Cancer Treatment

Treating OC using traditional chemotherapy has serious limitations, including the rapid clearance of drugs, undesirable biodistribution, and adverse side effects. To minimize these limitations, researchers have focused on a variety of drug delivery systems (DDSs) with which to encapsulate anticancer agents so they can directly reach tumor cells. Many types of DDSs have been developed, such as liposomes, drug conjugates, microspheres, micelles, nanoparticles, implants, and injection depots [126]. The benefits of using a DDS over conventional chemotherapy include the lower nonspecific toxicity, increased exposure of cancer cells to the drugs, circumvention of drug resistance, and improved drug solubility.

In 1996, researchers published the first report on biodegradable and biocompatible nanoparticle compositions using poly(lactic-co-glycolic) acid. Various improvements and adjustments have been made to the material, and nanoparticle synthesis processes have been continually updated. Recently, there has been growing interest in employing naturally existing protein cages, such as viral particles, as drug carriers [127,128,129], while the majority of research has focused on designing nanoparticles for delivering chemotherapeutic agents such as cisplatin, doxorubicin, and paclitaxel as an advanced therapeutic option for OC [130]. The polymers most widely used in drug delivery systems include polylactic acid (PLA), polylactic-co-glycolic acid (PLGA), poly(γ-glutamylglutamine), polyethylene oxide, modified poly -ε-caprolactone (PCL), and polypropylenimine (PPI) [130,131,132]. In addition to designing diverse nanoparticle materials, it is possible to make various surface modifications to either sustain the controlled release of drugs or enhance drug stability [133]. 

### 5.1. Single-Agent Delivery Systems

To enhance the efficacy of cancer treatment, a minimum of one chemotherapeutic agent is encapsulated or embedded into nanoparticles. The drug cisplatin is widely used as first-line therapy for ovarian cancer, but it has a dose restriction due to its nephrotoxicity [134]. Therefore, researchers have made efforts to improve the distribution of cisplatin and reduce kidney damage by using surface modification and nanoparticle engineering techniques [135,136]. Polyisobutylene-maleic acid (PIMA) linked to glucosamine (GA) was used to generate cisplatin nanoparticles by forming platinum (Pt) complexes toward each monomeric unit at various polymer-to-Pt ratios [135]. 

The chemotherapeutic agent paclitaxel is widely used in combination with a therapeutic drug carrier, but the small molecule is hydrophobic in nature (DrugBank, DB01229). To overcome the obstacle of its low aqueous solubility, the clinical dosage is used with absolute ethanol, making a combination called Cremophor EL, which is physiologically and pharmacologically potent; however, it has been shown to cause severe acute hypersensitivity [137]. ABI-007 (Abraxane^®^), an alternative to Cremophor EL, was later developed to improve the solubility of paclitaxel [138], and an albumin-bound paclitaxel nanomaterial was approved by the FDA for treating different types of cancers [139]. It is now a feasible alternative to paclitaxel in Cremophor EL drug formulations. Feng et al. developed nanoparticles comprising paclitaxel joined to PGG via an ester bond [140].

In addition to breast cancer treatment, the chemotherapeutic drug doxorubicin (Dox) is also extensively used in ovarian cancer, but it presents serious cardiotoxicity. To lessen its toxicity, doxorubicin could be encapsulated and delivered via a drug delivery system. Zeng et al. developed a naturally occurring biological scaffold for synthesizing doxorubicin-releasing nanoparticles by infecting the cucumber mosaic virus (CMV) [141].

### 5.2. Co-Delivery Nanoparticles

To achieve superior efficacy, particularly in chemotherapy, and minimize the toxicity of single-drug therapy, nanodrug co-delivery systems (NDCDSs) have been developed, using combinations of at least two anticancer drugs with different physicochemical and pharmacological properties [142]. It is possible to incorporate drugs, antibodies, and siRNA into the nanoparticles, facilitating the administration of numerous drugs in a single dose. For example, paclitaxel and ceramide were co-delivered utilizing PEO-PCL nanoparticles [143,144]. Ceramide buildup within cancer cells induces apoptosis and enhances the effectiveness of chemotherapy. However, ceramide cannot be administered to the systemic circulation due to its hydrophobicity, limited cell permeability, and metabolic inactivity. Therefore, biocompatible and biodegradable nanoparticles with paclitaxel and ceramide co-delivery were developed for effective ovarian cancer treatment.

There have also been significant developments in siRNA-based drug co-delivery systems. By using polypropylenimine (PPI), a new dendrimer that efficiently transported paclitaxel and a siRNA specific for the CD44 mRNA was synthesized [145,146,147]. CD44, a glycoprotein present on the membranes of cancer cells, plays an essential role in cancer development and progression. It was expected that the siRNA-mediated inhibition of the cell surface CD44 marker would prevent the development of metastasis and improve the efficacy of chemotherapy treatment. A delivery vehicle was developed to overcome the slow penetration of siRNA into the cell membrane. A polypropylenimine (PPI) dendrimer was designed along with the chemotherapeutic drug paclitaxel to deliver siRNA for CD44 suppression. [146]. However, the issues of biodegradation, bioavailability, instability, tissue distribution, and possible toxicity raise concerns about their safety for long-term administration [148]

## 6. Limitations and Chemoresistance of Ovarian Cancer Therapy 

More than half (58%) of OC patients are diagnosed at an advanced stage (III or IV), which prevents early diagnosis and leads to poor prognosis [149]. The standard of care for advanced OC includes cytoreductive tumor surgery followed by chemotherapy and/or radiotherapy regardless of tumor heterogeneity, hormone therapy, etc. [150]. However, chemotherapy resistance is still considered a major challenge when attempting to cure patients and achieve a favorable prognosis because the exact treatment choice depends on a number of factors, including the cancer molecular subtype, stemness, and clinical stage; the disease dynamics; and the person’s age and overall health [151].

A variety of chemotherapeutic agents for treating ovarian cancer that can be used singly or in combination are available [13,152]. The most commonly used chemotherapeutic agents are platinum-based drugs (cisplatin and carboplatin) and taxane family drugs (paclitaxel and docetaxel) [153]. Unfortunately, these agents are associated with different types of life-threatening side effects, including sustained nausea and vomiting, hair loss, mouth sores, acute renal injury, ototoxicity, infertility, anemia, leukopenia, thrombocytopenia, and long-term peripheral neuropathy [9]. In fact, chemotherapeutic agents have poor bioavailability, high dose requirements, low therapeutic indices, and nonspecific targeting, which ultimately lead to elevated toxicity in normal cells and drug resistance in cancer cells [148].

Chemotherapy resistance is a complex phenomenon in which cancer cells evade the effects of chemotherapeutics. Multidrug resistance (MDR) is considered the main cause of chemotherapy treatment failure and low patient survival rates [154]. With MDR, cancer cells become insensitive to both cytostatic drugs and pharmaceutical agents. The resistance emerges rapidly through multiple mechanisms such as drug inactivation, alterations in the drug target, drug efflux (e.g., P-glycoprotein), DNA damage repair, the evasion of apoptosis [154], the activation of drug-metabolizing enzymes (e.g., cytochrome P450 and glutathione S-transferase) [155], and genetic (gene mutation and amplification) and epigenetic (methylation and acetylation) changes [154]. Among these mechanisms, some favor drug resistance by reducing the effective concentrations, while others contribute by inhibiting the toxic action of the drugs [156]. However, due to advancements in DNA microarrays, proteomics technology, and the development of novel targeted therapeutics, new strategies for overcoming drug resistance can be provided. 

Radiotherapy has been used extensively for the treatment of dysgerminomas and the clearance of residual malignancy after surgical removal. However, despite the therapeutic effects with regard to the clinical management of ovarian cancer, the development of resistance is apparently unavoidable, which impedes further treatment [157]. Therefore, understanding the underlying molecular mechanism of therapeutic resistance is crucial in the management of ovarian cancer and drug discovery, which will improve clinical outcomes. 

## 7. Technological Advances in Identifying Novel Biomarkers of Ovarian Cancer

Despite the widespread use of traditional and modern technology for the detection and prognosis of OC, it remains the deadliest gynecological malignancy in terms of early diagnosis and management [150]. Therefore, it is urgent to search for novel diagnostic and prognostic biomarkers of ovarian cancer in order to understand the disease’s biology, which could provide guidance for improved treatment decisions. Currently, multi-omics approaches (genomics, transcriptomics, proteomics, and metabolomics) provide unprecedented opportunities to understand disease pathophysiology at different molecular layers, which can facilitate the accurate prediction of disease biology. The molecular markers identified by these approaches are crucial for disease prognosis by predicting tumorigenesis, progression, and metastasis, based on the continuous improvement of the technologies [16,17,158,159,160]. The discovery of novel biomarkers could guide targeted therapeutic decisions by accurate prognostication, thereby minimizing unwanted side effects and therapy resistance, which could improve the management of ovarian cancer toward achieving a better quality of life and patient survival outcomes (Table 1) [161,162,163].

In genomics, oncogenes, tumor-suppressor genes, and epigenetic modifications of DNA can be detected at the DNA level through gene mutation and DNA methylation microarrays, genome-wide association studies, and sequencing [163,167,175]. The mutation of TP53 is the most frequent genetic abnormality; it causes loss of function in OC and is demonstrated in 60–80% of patients in both sporadic and familial cases [4]. DNA repair defects were found in 10–15% of ovarian cancers; the lifetime risk for BRCA1 is about 30–60% and that for BRCA2 is 15–30% in those who have a genetic defect promoting the development of OC [4]. In addition, the epi-biomarkers RUNX3/CAMK2N1, ARNTL, and Fkbp1/Pax9, detected by GWA, CpG island microarrays, ChIP-PCR, and Sanger sequencing, can predict prognosis, clinical outcomes, and chemotherapy resistance [161,162,167].

In transcriptomics, coding mRNA and ncRNA microarrays and RT-qPCR are widely used to explore disease biology and dynamics with a comprehensive assessment of changes in expression patterns by observing the differential expression of genes and differently spliced transcripts at the RNA level, including mRNA, miRNAs, lncRNAs, and circRNAs in ovarian cancer (Table 1). In some cases, high collagen type XI alpha 1 (COL11A1) expression at the mRNA level is associated with advanced disease stage, recurrence, and poor survival via the TGF-β1–MMP3 axis and pathways [170]. The forkhead box M1 (FOXM1) oncogene is upregulated (mRNA) in EOC; it is involved in cell cycle progression predominantly through the regulation of cell-cycle-checkpoint genes and is a potential prognostic biomarker for chemoresistant OC [163]. The circCELSR1, a circular RNA (circRNA), was found to be dramatically upregulated in PTX-resistant OC, as determined by microarray analysis and quantitative real-time PCR, dual-luciferase reporter assays, and RNA immunoprecipitation, and the circCELSR1–miR-1252–FOXR2 axis was finally established as a novel therapeutic target in OC [158].

Regarding protein levels, differentially expressed proteins, antibodies, cytokines, growth factors (proliferating and proangiogenic factors), etc., could be very useful in the early diagnosis and prognosis of OC through high-throughput techniques such as LC-MS, ITRAQ tagging coupled with mass spectrometry, reverse-phase protein arrays, etc. [16,19,172,173]. For example, serotransferrin, AA1, Hpx, CRP, and albumin, found to be differentially expressed in OC, can be used in a multimarker test for the screening and detection of ovarian cancer [16]. Retinol-binding protein 4 (RBP4) is an adipocyte-derived cytokine that contributes to the pathogenesis of endometriosis by increasing the viability, proliferation, and invasion of endometrial stromal cells [172]. Therefore, novel efficient diagnostic platforms are needed to detect OC biomarkers with high sensitivity and selectivity, miniaturization, versatility, and high throughput. The identification of new biomarkers for early diagnosis is also required in order to increase the survival rate and quality of life of ovarian cancer patients.

## 8. Conclusions

Ovarian cancer is a deadly gynecological illness that affects women worldwide. Due to a lack of precise diagnostic biomarkers, the majority of women with ovarian cancer are diagnosed at an advanced stage, which reduces their chances of survival. Chemotherapy resistance in late-stage ovarian cancer is a significant clinical challenge, because various signaling pathways are involved in the pathophysiology of chemotherapy resistance. In order to address this, the focus is on developing biomarkers and diagnostic tools that can help with the early detection and prediction of the disease. It is hard to determine the molecular changes occurring in ovarian cancer, which is very important for choosing the right therapeutic drugs, the success of which can improve clinical outcomes. Thus, it is critical to understand the biology of this heterogeneous disease in order to conduct more precise investigations into its mechanisms. Advancements in our understanding of ovarian cancer biology has resulted in the identification of a variety of molecular targets, including signal transduction pathways, growth factor receptors, angiogenic processes, and cell cycle regulators, as well as drug delivery systems. In addition, advances in therapeutic technology have allowed significant insight into the molecular complexity, creating opportunities for diagnosis and prognosis to inform new therapeutic efforts which have the potential to significantly improve the overall survival rate and quality of life of patients with ovarian cancer.

## Figures and Tables

**Table 1:** Emerging prognostic biomarkers in ovarian cancer and novel technologies.

Biomarker/ Drug/ Inhibitor	Treatment Strategies/ Components	Therapeutic Response	Features/ Properties/ Nature	Detection Level	Supported Technologies	Refs.
PARP inhibitor	Extended PFS	OC, phase 3 trial	Personalized medicine	HRD-positive tumors		[164,165,166]
PARP inhibitor, bevacizumab	PFS benefit, anti-VEGF	OC, phase 3 trial	Antiangiogenic	HRD-positive tumors, BRCA mutation		[100]
Combination of PARP and ATR inhibitor	Overcomes PARPi and platinum resistance	OC, PDX models	Stabilize stressed replication fork and apoptosis	DNA, protein	Western blot, IHC, NGS, RPPA	[17]
ARNTL	Epi-biomarker by reducing promoter methylation	OC	Circadian and tumor-suppressor gene	DNA	CpG island microarray, COBRA, ChIP-PCR	[167,168]
RUNX3/CAMK2N1	Epigenetic prognostic marker	EOC	Hypermethylation of CpG island reduces PFS	DNA	GWA and targeted NGBS confirming array	[162,169]
Fkbp1/Pax9	Epi-biomarker for platinum-resistant therapeutic target	OC	PAX9 hypermethylation causes a poor prognosis for OS	DNA, RNA	Sanger sequencing, RT-PCR	[161]
COL11A1	Promotes tumor progression through TGF-β1–MMP3 axis and predicts poor prognosis	OC	Disease-progression-associated gene	mRNA	Microarray, RT-PCR, casein zymography, and ChIP assay	[170]
circCELSR1	Increases paclitaxel resistance and poor prognosis	OC	Circular RNA	miRNA	Microarray analysis and RT-qPCR	[171]
microRNA-137	Promotes apoptosis; represses mRNA translation	Improves drug resistance	Regulating RNA	Short non-coding RNA	Dual-luciferase reporter assay	[158]
FOXM1	Prognostic and chemoresistant therapeutic target	Non-serous EOC	Oncogene	mRNA, protein	Microarray, RT-qPCR, and IHC	[163]
RBP4	Diagnostic or prognostic biomarker	Ovarian endometrioma	Adipokine RBP4 involved in the pathogenesis of endometriosis	Protein	Human XL proteome profile assay, IHC, cell viability, and invasiveness assay	[172]
AAT, NFKB, PMVK, VAP1, FABP4, and PF4	Predicts prognosis	HGSOC	Differentially expressed proteins	Protein	Hierarchical clustering, bioinformatics, LC-MS, and IHC	[19]
Serotransferrin, amyloid A1, hemopexin, C-reactive protein, albumin	Multimarker test specific for screening and detection of OC	OC	Molecular signaling pathways of OC	Protein	ITRAQ-tagging coupled with mass spectrometry	[16]
PDGFR-beta and VEGFR-2	Predictive biomarker for treatment response	OC	Angiogenesis-related growth factor receptors	mRNA Protein	Quantitative RPPA, bioinformatic analysis	[173]
Circulatory protein	Personalized therapy for early diagnosis and prediction of drug resistance	OC	Proteomic landscape	Protein	Proteomic	[174]
2-piperidinone and 1-heptadecanoylglycerophosphoethanolamine	Clinical diagnosis and treatment	OC	Candidate biomarker	Metabolites	UPLC/Q-TOF MS	[159]

## Data Availability

Not applicable.
